# The Nordic maintenance care program: what is maintenance care? Interview based survey of Danish chiropractors

**DOI:** 10.1186/2045-709X-21-27

**Published:** 2013-08-20

**Authors:** Corrie Myburgh, Dorthe Brandborg-Olsen, Hanne Albert, Lise Hestbaek

**Affiliations:** 1 Department of Sports Science and Clinical Biomechanics, University of Southern Denmark, Odense, Denmark; 2 Nordic Institute of Chiropractic and Clinical Biomechanics, Campusvej 55, Odense 5230, Denmark; 3 Research Department, Spine Centre of Southern Denmark, Institute of Regional Health Services Research, University of Southern Denmark, Odense, Denmark

**Keywords:** Maintenance care, Chiropractic, Prophylaxis, Prevention, Health care services, Public health

## Abstract

**Objective:**

To describe and interpret Danish Chiropractors’ perspectives regarding the purpose and rationale for using MC (maintenance care), its content, course and patient characteristics.

**Methods:**

Semi-structured interviews were conducted with 10 chiropractors identified using a stratified, theoretical sampling framework. Interviews covered four domains relating to MC, namely: purpose, patient characteristics, content, and course and development. Data was analysed thematically.

**Results:**

Practitioners regard MC primarily as a means of providing secondary or tertiary care and they primarily recommend it to patients with a history of recurrence. Initiating MC is often a shared decision between clinician and patient. The core elements of MC are examination and manipulation, but exercise and general lifestyle advice are often included. Typically, treatment intervals lie between 2 and 4 months. Clinician MC practices seem to evolve over time and are informed by individual practice experiences.

Chiropractors are more likely to offer MC to patients whose complaints include a significant muscular component. Furthermore, a successful transition to MC appears dependent on correctly matching complaint with management. A positive relationship between chiropractor and patient facilitates the initiation of MC. Finally; MC appears grounded in a patient-oriented approach to care rather than a market-oriented one.

**Conclusion:**

MC is perceived as both a secondary and tertiary preventative measure and its practice appears grounded in the tenet of patient-oriented care. A positive personal relationship between chiropractor and patient facilitates the initiation of MC. The results from this and previous studies should be considered in the design of studies of efficacy.

## Background

Maintenance care (MC) is used by chiropractors to treat patients who are no longer in an acute state of pain; the purpose being to prevent recurrence of episodic conditions (secondary prevention) and/or maintain a desired level of function (tertiary prevention). The concept is frequently used among chiropractors [1,2] and limited evidence suggests that, among workers with work-related back pain, MC in chiropractic practice appears to decrease the recurrence rate [3]. However, according to two literature reviews, very limited evidence regarding the definitions, purpose and content of MC is currently available [4,5].

As a result, several investigations aimed at increasing and clarifying information on MC have been launched. Specifically, investigators involved with the Nordic Maintenance Care Program have conducted a number of observational, questionnaire-based and qualitative studies, in relation to MC practices for low back pain. The results, thus far, have proven useful in increasing knowledge regarding issues such as usual time intervals between MC treatments [2,6], treatment strategies for different back pain scenarios [1,7,8], the content of MC consultations [6] and patients perception regarding the purpose of MC [6].

Furthermore, the effect of MC has been investigated in clinical trials with varying results. A pilot study included low back pain patients [9] and two randomized controlled trials (RCTs) included chronic non-specific neck pain and chronic non-specific low back pain, respectively [10,11]. However, in all three studies patients were included consecutively based on neck or back pain alone, but apparently without taking into account the underlying rationale for MC in the inclusion process.

An argument can be made that there is still a need for a more in-depth understanding of the chiropractic professions’ own view on the concept of MC, before investigations progress to the level of RCTs. We therefore conducted a further qualitative investigation, in this instance focusing on the care provider. Specifically, we focused on Danish chiropractors, in order to develop a further understanding of their perspectives regarding the purpose and rationale for using MC (maintenance care), its content, course and patient characteristics.

## Method

### Design

A phenomenological case study was deemed appropriate to observe the conceptualization of MC in a private practice context.

### Units of observation and sampling framework

Although MC practices can be observed from multiple perspectives, our focus was on the perspective of the service provider (chiropractor).

A background/reference group was set up consisting of 6 chiropractors, all clinicians, who were known by the research group to use MC in their everyday practice. This group participated in the design of the present study and also, at a later stage, during the analysis of results.

### Selection of study participants

Two chiropractors from each of the five regions in Denmark were contacted telephonically and asked to give their opinion on whether peers, practicing in their region, used maintenance care (MC) to a low (<20%), medium (20-50%), or high degree (>50%). The cut-points were based on observations previously made regarding maintenance care practices in the Scandinavian context [1]. It was stressed that their opinions would anonymous. The sample was furthermore stratified to take into account country of education as previous investigation in the Danish context indicated that the MC utilization seems to relate to the country chiropractor was educated in [1]. Most chiropractors practicing locally are educated in the USA, England or Denmark itself. Thus, we also included at least one chiropractor from each of the three countries. Our target sample of nine respondents (n = 9) was also stratified to take into account gender and to have an adequate geographical spread. Finally, we included a tenth respondent to ensure that data saturation would be reached.

The targeted chiropractors were contacted by telephonically and given a brief introduction to the project. They were also asked in which group – high, medium or low use of MC – they would place themselves. If peer- and self-perception aligned, the individuals were asked to join the study; non-alignment was therefore an exclusion criteria.

### Interview guide

A semi-structured interview schedule was constructed based on the previous literature [1,2,6,7,12,13] and input from the background group (see Additional file 1). The schedule was designed to emphasize the collection of data relating to four overall domains:

1. Purpose: the rationale and the benefits of MC practices.

2. Patient characteristics: the type of patients believed to benefit from MC.

3. Content: a description of the MC-consultations.

4. Course and development: Initiation and termination of MC as well as spacing between visits.

A semi-structured interview was deemed most appropriate, because it allowed for free and open responses within the broad framework of MC already established.

### Pilot procedure

Prior to the study two test-interviews were conducted, to establish time duration requirements and ensure the clarity of question posed. The information obtained was not analyzed as part of the present study.

### Trustworthiness of data

In order to establish and maintain trust between the interviewer and respondents three aspects were considered, these being that both the interviewer and the rest of the research team were chiropractors and thus part of the same profession as respondents, the explorative and neutral nature of the project was emphasized and respondents were privy to the MC rating assigned them by colleagues and were aligned to this (low, medium or high) and therefore their own role in the project was clear and accepted.

### Interview procedure

As an introduction to each interview the interviewer made it clear that it was of great importance that the chiropractor felt that he/she was given the opportunity to elaborate freely on his view on MC. We used the systems CHILDES (Child Language Data Exchange System, http://childes.psy.cmu.edu/) and CLAN (Computerized Language Analysis) for digitalized recording after which sound files were then exported for verbatim transcription. The transcriptions were conducted by the interviewer (DO). This study was deemed exempt from ethics approval, however, study was conducted in compliance with the stipulations of the Danish Data Protection Agency with respect to the procurement and storage of anonymous interview data.

### Analysis

**Figure 1:**
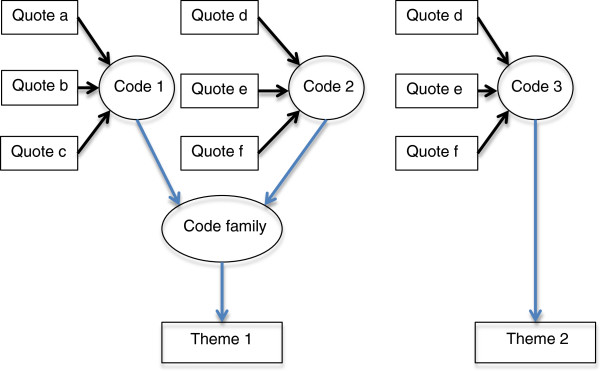
Conceptual framework for the categorization and synthesis of data during qualitative analysis.

Our conceptual framework for the categorization and synthesis of data is presented in Figure 1. It is important to note that a single quote can contribute several different codes. Codes may be found to have a relationship with one another, either through shared quotes or inferred meaning, thus resulting in the development of code families. Finally, several code families may contribute to a theme or a single code may be raised to the level of a theme.

### Sorting

Manual coding was used throughout this study. Interview one was coded independently by the primary interviewer (DO) and a naïve, second checker (HA); both used an inductive approach and generated a code list, which was merged by means of discussion and consensus.

### Categorizing

Using the merged code list, two further interviews were coded independently, during which time code families were also generated. As with the codes, consensus was similarly reached regarding the resulting code families. The remaining seven interviews were coded by the primary interviewer.

### Synthesis

Finally the code families (and the codes they contained) were related back to the domains of MC in the form of a matrix, so that meaning could be extracted in the context of the MC encounter.

When saturation was reached, the chiropractors from the background group were invited to a meeting in order to confirm saturation. All the extracted codes were presented and the chiropractors were asked if they were able to recognize their own opinions among the presented codes and whether they had anything to add.

## Results

**Figure 2:**
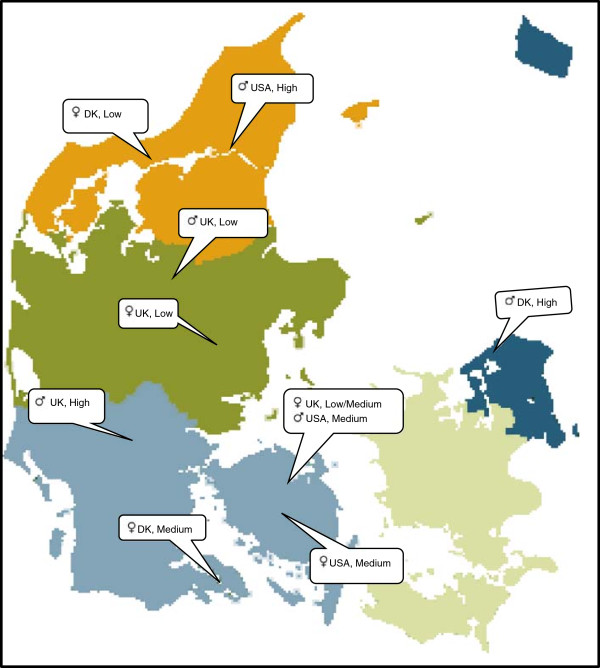
Profile of study participants indicating geographical spread, location, gender, country of education and level of maintenance care usage.

All of the practitioners sampled agreed to participate in the study. The profile of our sample is illustrated visually in Figure 2. Our tenth respondent was rated as a borderline low/medium level MC practitioner, both by her peers and by herself. Interviews were conducted over a two-month period, typically lasting between 20 and 50 minutes.

### Data saturation

No new statements or points of views were noted after interview 10. Furthermore, the chiropractors in the background group all felt that their points of views were covered and had nothing further to add. We were therefore satisfied that data saturation had in fact been reached and no further interviews were conducted.

### Thematic analysis

As our analysis developed, it became apparent that the creation of code families would be an unnecessary duplication. We consequently identified three main themes from the responses, these being ‘The rationale and motivation of maintenance care’, ‘The maintenance care model’ and ‘significant professional considerations’ (see Additional file 2).

#### The rationale and motivation for maintenance care

We observed two key conceptual frameworks motivating practitioners to provide MC.

The most dominant is the notion of MC as a preventative practice. Specifically, the provider aims to stop/curb recurrence. This is considered achievable by combining treatments, such as manipulation and other manual interventions with patient education regarding ergonomic hygiene, exercise and healthy living:

“…if I can hit the point where as many as possible are functioning as good as possible for as long as possible – that’s what makes me feel I’m doing something right…”

“…I actually think I experience a lot of cases where you can help patients who used to have 3-4-5 cases of low back pain a year – they don’t have that when they get MC…”

Aligned with this is the ‘lifelong chiropractic for all’ ideal of maintaining a certain level of attained function for the patient:

…if we check them once, twice maybe three times a year – look, no problems! My goal is to fix the initial problem and then attend to it regularly afterwards to keep it working”

A less common perspective espoused is that MC represents the core motivation for providing treatment:

…if we use the degenerative joint disease model the aim is to keep those joints as freely moving as possible, as [straightened out and well-functioning] as possible, at as good a posture or position as possible and have as strong and well-coordinated muscles as possible”

and

The body is built to heal itself if it is given good nutrition, good sleep, exercise, clean air and water…it can manage all of this if we make sure that the nervous system is working right

MC practices appear to be grounded in two key considerations, these being the maintenance of an acquired improvement and/or the prevention of recurrence:

…there is the MC where the patient doesn’t have any symptoms, but out of fear of getting symptoms…or because they wish to function optimally they come in…so in this way it isn’t symptom based treatment”

…the other category is guided by symptoms, they feel that they are getting a little more pain…it’s time for me to go and get a treatment… often this is a person whom we can’t get a 100% on top”

However, there is also a perspective that the MC practices stop the occurrences of symptoms stemming from sub-optimal nervous system function.

Furthermore, it appears that at least three determinants for initiating care are observable namely; fear of recurrent symptoms, a perception of sub-optimal biomechanical and the return of to self-reported patient outcomes, such as pain.

#### The maintenance care model

##### Who is a potential MC patient?

Our respondents identified seven factors that may help to profile a MC patient candidate. As indicated in Table 1, these individuals are unlikely to be children presenting with a first episode of back and/or neck pain.

**Table 1:** **Who might be an MC candidate patient**?

**Unlikely**	**Could be**
• The acute or ‘first episode’ patient	• A past history of chronicity and/or extended courses of treatment
• Peadiatric patient	• More likely to be female
	• Patients complaining of ‘muscular’ trouble (e.g. tension)
	• Occupations where taxing physical labor form a predictable part of the working day.
	• Biomechanical/structural variant (e.g. scoliosis)

Perhaps of more interest, however, was our observation that ‘more often than not it’s simply a question of testing out which strategy seems to fit the single patient the best’. Thus, for those practitioners who actively attempt to determine whether a patient is likely to benefit from MC, considering the level of clinical interaction and the personality of the patient is important. It is therefore more likely that the MC patient is one who has developed a relationship with the chiropractor:

…we are not just machines…..it’s quite alright to have some personal relation to a certain degree, and this is particularly the case with the MC patients.

In some instances, where patients are considered to be in process of developing a dependence on treatment, MC is initiated as a strategy to control/limit the frequency of care:

[I am]…tired of those who think they are to come here once a month…they are not allowed to do that

…I also have patients who come more often than I think they should…you try to increase the interval between treatments all the time but…”

Profiling the MC patient was, however, not an important consideration for all respondents. These were respondents who presented the poles of the MC spectrum, namely those who considered MC universally beneficial to every patient:and those who typically try to avoid it:

…the majority of patients in the clinic, that’s what I aim for.

…everyone with a normal sensory capacity feels that this feels good…..and that, I think, is what sells it.

…[I only give it] if the patient directly asks for it.

##### Initiating and transitioning into MC

It appears that practitioner, patient or both initiate MC. Practitioners may use a suggestive strategy to introduce the notion depending on the patient’s initial problem and history. For example:

…if your goal is to do the best possible for yourself (the patient) and get in as good a shape as possible then we (the chiropractor) can help you to do so, and if you want to be checked up upon once in a while we can do that too”…We always let them sort of decide.

However, a concrete recommendation approach can be followed such as “now listen we have to address this far more consequently as well with treatment as training!”

Patients on the other hand appeal to the practitioner with statements such as “I don’t think I can manage without some treatment, I would like to come on a regular basis.”

In instances where a joint decision to initiate MC is made agreement is typically reached that “…it might be a good idea to try that strategy for a while.”

Depending on the strategies mentioned above, the transition point to MC may vary, but usually MC commences when treatment has been well established:

…if we get to the point where things are actually working, no or only limited symptoms, ok let’s see how it works in a month, 2 months, 3 months and find out what interval keeps you working.

And

… well, you can say it’s something I do straight away…to tell them what I do….”now that my pain has gone why should I continue to come?” …I try to tackle and address that straight away.

Whilst initiating and transitioning a patient into MC varied considerably depending on the clinician’s individual circumstances and practice exposures, MC practices nevertheless appear to evolve over time.

…I had no experience with MC whatsoever when I started.

…it was not at all on the schedule at school; we didn’t even talk about it!

…in the beginning it was probably the patients themselves who said – “can I come on a regular basis?” – now it’s more me who suggests that it might be a good idea.

…I just found out myself that it works…

…the time spent in the same clinic for my part has been of essence…..to be able to follow the same persons for years

…I took over a clinic where a lot of MC treatment was given; too much!

However, MC also appears to reflect individual perspectives relating to disease-oriented and holistically oriented service provision:

…do you really want to do something about it [the cause of your problem] or do you just want momentarily relief of your pain?

and

…it makes up a package. I don’t think it makes sense to only consider peoples back problems, you have to take into consideration how they live, right?

##### Content of MC

Our group of respondents provided a rather heterogeneous description of the content of MC; these varied from so-called ‘pure chiropractic help’, consisting of examination and if necessary manipulation to patient specific ‘packages’ including elements such as exercise prescription and actual training, guidance on ergonomics, diet, weight loss and stress management. The role of strength and conditioning training, in particular, appeared to be a cause of disagreement, with respect to its inclusion as part of MC:

…those I consider to have a great risk of recurrence I would probably recommend to work out on a regular basis instead of suggesting MC.

Despite the apparent incoherence of content, a fundamental part of MC appears to hinge on providing the necessary attention and care for the patient, which is achieved by motivating and helping patients maintain focus on beneficial habits and lifestyles:

…to help them remember what they can do themselves…that’s a big part of the MC treatment

##### Frequency and termination of MC

Our observations suggest that treatment interval is determined on a case-by-case basis; with factors such previous injuries, age, body-type, recurrence, and the presence of degenerative joint disease informing the clinician’s ‘*sixth sense*’. Periodicity ranges between 2 to 4 months, typically evenly spaced over a calendar year.

When initiated by the patient, however, frequency coincides with the patient’s sense of ‘control’ of their particular problem:

…upon solution of their initial/acute problem I have told quite a few patients that I find it beneficial for them to get into MC treatment, and they respond that they feel confident that they can control it themselves and wish to call the clinic when they get any of their well-known symptoms…

Respondents did not appear to experience tension with patient-determined intervals ‘… if they are able to react on their symptoms before a regular relapse.’ However, they were mindful that this mode could increase treatment frequency.

Our observations indicate that MC is terminated when symptomology is absent for extended periods. Cost on the other hand may not be a primary driver for cessation:

…even if they get their treatments for free it is my experience that they don’t want more treatment than they actually need.

And

…even though I have patients who get 100% insurance paid treatment ….. I don’t think they would come here for no reason…

#### Significant professional considerations

Considering one’s professional ‘conscience’ whilst planning a course of treatment emerged as an important theme associated with ‘the purpose and rationale of MC’. There was broad agreement among the practitioners interviewed that MC should not be a standard choice and that it should not be offered to or used for every patient:

…maintenance is an individual solution…this is part of our unique product.

…one should not pressure patients into MC treatment if they have no need and I think that some people don’t have that need…

…we can’t just tell people to come back once a month. I know a lot of chiropractors around the world [do this]… their maintenance programs are 3–4 weeks regardless…

Furthermore, patients who receive MC should have a real need and that they feel a benefit from getting the treatment:

…it must not just be a convenient thing for me, that’s not right in an ethical sense, not even if people ask for it themselves, there has to be an actual and real need…

…coming in every three months on a regular basis without having symptoms… I can’t make myself practice that way…

Interestingly the awareness that MC can potentially be misused as a strategy to justify over servicing, appears to have resulted in some respondents declining MC even if their perception was that the patient would benefit:

…clearly, some [DC’s] will do it [MC] out of economical reasons alone ,others will find that this is not okay and then they forget that there might be other reasons than money to place patients on MC..

… it may very well be that I “cheat” some of my patients…. I can’t deny that some of my patients might benefit from MC but don’t get it…

Our respondents were aware that positions vary with respect to MC and that it is a source of tension for the profession. This position is best voiced in the following short discourse:

…some are kind of very much against the use of MC and some are very much for it…but on the bottom line there are patients who wants one thing and patients who wants another so it seems reasonable enough to offer different things…

…it’s very much those who don’t use MC… they are almost angry of those who do….but on the other hand some of the MC chiropractors think that the others simply let down their patients for instance when they don’t bother to have training facilities . I for one find that absolutely wrong.

Regardless of individual position, our respondents considered it important that MC practices be disassociated from a market-oriented strategy for building and maintaining a practice in the Danish context. In this regard they state the following:

…it’s “un-Danish” this thing to plan long schematic treatment courses…

…I don’t want to be known as someone where it’s said that – “you have to come here for the rest of your life” … I have seen Mr. XXX himself in England… Completely unethical if you uncritically plan for every patient to come back for many many treatments…

…it has to be based on the given situation….otherwise we risk ending up as in USA where 60 treatments can easily be planned on your first visit….

## Discussion

Our investigation revealed clear themes confirming previous results from quantitative studies [1,2,6-8,12,13]. In particular, as seen in other contexts, Danish chiropractors regard MC primarily as a means of providing secondary or tertiary care, it is recommended to patients with a history of recurrence, the initiation of MC is often a shared decision between chiropractor and patient, the use of MC relates to the chiropractor’s education and clinical experience, the core element of MC is examination and manipulation, but also commonly includes exercise and general lifestyle advice and finally the typical interval between consultations is 2–4 months.

In addition to these confirmatory observations our investigation also revealed that in the local context chiropractors were more likely to offer MC to patients whose complaints included a significant muscular component, that a successful transition to MC appears dependent on a correct matching of complaint and management, that a positive relationship between chiropractor and patient facilitates the initiation of MC, and finally that MC rests in the tenet patient-oriented care rather than market-oriented. Previous studies have indicated that the patient needs to respond positively to chiropractic care before MC is offered, but this issue did not emerge in the present study. Whether this is because the interviewed chiropractors did not find it important, or whether they considered it obvious and therefore didn’t mention it, is unknown.

We selected as broad a range of Danish chiropractors and conducted enough interviews to reach the point of saturation. We also believe our data to be trustworthy due to the nature of the interviews and the apparent trust between the interviewees and the research team. However, the nature of this type of investigation precludes us from inferring a generalizable truth about MC in Denmark. Nevertheless, these results are synergistic to other investigations on this topic and as such it seems fair to consider results from the present study as relevant indicators for the Danish chiropractors’ view on MC.

The concept of MC as secondary or tertiary prophylaxis varies somewhat from the most common perception of prophylaxis, which focuses on primary prevention, i.e. to prevent disease from occurring. Typical examples of the latter include vaccines for communicable diseases or condoms to avoid sexually transmitted diseases (STDs), where the purpose is primary prevention. However, in public health, many initiatives aimed at primary prophylaxis also function as secondary or tertiary prophylactics. For example physical exercise may prevent cardiovascular disease; however, it also serves to regulate blood pressure after the onset of the disease.

MC practices are not unique to the chiropractic profession. In dentistry, MC is intended to avoid caries and periodontal disease through fluoride therapy and improved dental hygiene. However, if such disease occurs despite the primary prevention strategy, secondary preventative treatment takes effect to avoid exacerbation. Thus, the concept is generally recognized in society in other health domains and the implicit overall aim is to decrease the burden of disease and thereby also reduce the cost of health care.

Low back pain is now the leading cause of disability globally measured in years lived with disability (YLD) with 1206 YLD per 100,000 in 2010 and neck pain is number four with 488 YLD per 100.000 (GBD 2013). This represents an increase of 33.3% since 1990, largely driven by population growth and ageing [14]. Thus, these figures are likely to continue to increase. Parallel to this, there has been a significant increase in the consumption of painkillers, i.e. the sale of opioid analgesics has quadrupled between 1999 and 2010 [15]. Considering that more than 100.000 deaths per year can be attributed to adverse effects of medication in the US alone [16] as well as non-quantifiable morbidity, non-pharmaceutical prophylactic strategies deserve attention and for the musculoskeletal system chiropractic care might be an option. Limited evidence is currently available with respect to the effectiveness of MC strategies initiated by chiropractors. As stated previously, the RCTs available have included consecutive patients without consideration of either factors qualifying patients for MC in practice or individual care requirements. Therefore, these RCTs are unlikely to reflect clinical reality, and we suggest that investigators consider such factors and requirements in future studies, especially when planning RCTs.

## Conclusion

MC is a common phenomenon in Danish chiropractic practice, considered as both a secondary and tertiary preventative measure and its practice appears grounded in the tenet of patient-oriented care. A positive personal relationship between chiropractor and patient facilitates the initiation of MC. However, successful transition to MC appears to be dependent on a correct matching of complaint and management strategy. Interestingly, chiropractors in this study were more likely to offer MC to patients whose complaints include a significant muscular component.

It remains to be investigated whether MC is actually effective, both for the individual patient and in a societal/economic perspective. This is necessary in order to establish the appropriate role of MC in modern healthcare. The results from this and previous studies should be considered in the design of such studies.

## Competing interests

The authors declare that they have no competing interests.

## Authors’ contributions

DBO and LH contributed to conception and design of the study, DBO conducted the interviews, DBO, HA and CM did the analyses. LH and CM were responsible for the interpretation of the data. CM and LH drafted the manuscript. All authors read and approved the final manuscript.

## Supplementary Material

Additional file 1 Interview guide. Click here for file

Additional file 2 Codes and Quotes table. Click here for file
