# Supplementary material for: The Nordic maintenance care program: what is maintenance care? Interview based survey of Danish chiropractors
Source: Chiropr Man Therap. 2013 Aug 20;21:27. doi: 10.1186/2045-709X-21-27 (PMC3766236; doi:10.1186/2045-709X-21-27)
Supplement: Additional file 1 — Interview guide. [file 2045-709X-21-27-S1.docx]

**Appendix 1: Interview guide including English/Danish translation**

| ”Kategori”  Domain | **Hoved-spørgsmål:**  **Primary questions** | **Uddybende spgs./pkt der gerne skal belyses**  **Supplementary questions/ points that need to be covered** |
| --- | --- | --- |
| Intro/B | Jeg vil bede dig starte med at forklare mig hvad du forstår ved MC – din private ”definition”  I will ask you to start by explaining to me, what you consider MC to be – your private ”definition” | Indhold/elementer i MC forløb  +/- træning  +/- rådgivning etc.  Content/elements of MC courses of care  +/- exercises  +/- advice etc. |
| A | Når en patient behandles/ses i et MC forløb, har du så nogen bestemte generelle intentioner/forventninger til forløbet?  When a patient is treated in a MC regimen, do you have any specific overall expectations to the course? | Forbedret biomekanisk funktion  2° /3° forebyggelse  Improved biomechanical function  Secondary/tertiary prevention |
| (A), B, C | Kan du prøve at beskrive en typisk MC pt i din klinik for mig  Please try to describe a typical MC patient in your clinic | Forhistorie (past back history), køn, alder, problemstilling Previous history, gender, age, complaint  ”Behov” for god rygfunktion? Need for better back-function?  Hvor hyppigt kommer han/hun til behandling? What is the frequency of treatments?  Hvordan findes/fastlægges dette interval, er det fast eller fleksibelt fra gang til gang? How is the interval between treatments defined. Is it fixed or flexible from treatment to treatment? |
| (A), C | Overgangen fra ”alm” behandling til MC – hvordan kommer den i stand/på tale Transition to MC – how does it occur? | DC/pt induceret, fælles Is MC induced by the chiropractor, the patient or both?  Standard??? |
| C | Kan du fortælle lidt om hvordan eller hvorfor et MC forløb evt ophører?  Can you tell me how or why a course of MC can be terminated | DC/pt induceret, fælles, andet  Induced by DC, the patient, other |
| A | Oplever du nogen ændring i hvor meget ”efterspørgsel” der er på MC  Do you experience a change in the demand for MC? | Nogen relation til det øgede fokus på selvansvar i forbindelse med livsstilssygdomme+ Any relation to the increased awareness of patient responsibility in relation to lifestyle diseases.  Andre faktorer Other factors |
| A | Har dit syn på/opfattelse og brug af MC ændret sig væsentligt i løbet af din tid som kiropraktor - i givet fald hvordan/hvorfor  Has your perception and use of MC changed during your professional life – if so, how/why? | erfaring, ansat/selvstændig, turnus-sted/”læremester”, andet  Experience, selfemployed/employed, internship/”mentor”, other |

A: Formål og rationale/ Purpose and rationale

B: Indhold Content

C: Forløb/ Course and schedule
