# Supplementary material for: The Nordic maintenance care program: what is maintenance care? Interview based survey of Danish chiropractors
Source: Chiropr Man Therap. 2013 Aug 20;21:27. doi: 10.1186/2045-709X-21-27 (PMC3766236; doi:10.1186/2045-709X-21-27)
Supplement: Additional file 2 — Codes and Quotes table. [file 2045-709X-21-27-S2.docx]

| Theme | Code | Selected quote |
| --- | --- | --- |
| Rationale and Motivation for MC | Rationale | “…There is the MC where the patient doesn´t have any symptoms, but out of fear of getting symptoms… or because they wish to function optimally they come in… it isn´t symptom based treatment”  “The other category is guided by symptoms, they feel that they are getting a little more pain… now it´s time for me to go and get a treatment… often this is a person whom we can´t get a 100% on top [of]” |
|  | Motivation | “… If I can hit the point where as many as possible are functioning as good as possible for as long as possible – that´s what makes me feel I´m doing something right…”  “…I actually think I experience a lot of cases where you can help patients who used to have 3-5 episodes of low back pain a year – they don´t have that when they get MC…”  “… If we check them once, twice maybe three times a year – look, no problems! My goal is to fix the initial problem and then attend to it regularly afterwards to keep it working”.  “…If we use the degenerative joint disease model the aim is to keep those joints as freely moving as possible, … as good a posture or position as possible and have as strong and well-coordinated muscles as possible”  “The body is built to heal itself if it is given good nutrition, good sleep, exercise, clean air and water…it can manage all of this if we make sure that the nervous system is working right” |
| Course and content of MC | Who is a potential MC patient? |  |
|  | Who initiates MC? Are rationale and indications not two sides of the same coin? I like it, because it illustrates the secondary and tertiary prevention strategy | “…after the initial treatment part, we leave it up to the patient…. there are two ways to address it – you can either say okay, that was it, your symptoms are relieved, goodbye”  ”…if your goal is to do the best possible for yourself (the patient) and get in as good a shape as possible then we (the chiropractor) can help you to do so, and if you want to be checked up upon once in a while we can do that too”…..so we always let them sort of decide”  “…depending on their initial problem and their history I might suggest it (MC) to them”  “…now listen we have to address this far more consequent as well with treatment as training!”  “…I am the one who decides whether they should be given MC”  “…in the beginning it was probably the patients themselves who said: “Can I come on a regular basis?” Now it´s more me who suggests that it might be a good idea”  “…We kind of agree that it might be a good idea to try that strategy (MC) for a while”  “… if at a point of time they say:“I don´t think I can manage without some treatment, I would like to come on a regular basis” …they have a fair chance of getting MC if I agree on their point of view”. |
|  | Transitioning into MC? | “…if we get to the point where things are actually working, no or only limited symptoms, ok let´s see how it works in a month, 2 months, 3 months and find out what interval keeps you working”  “… well, you can say it´s something I do straight away…to tell them what I do….”now that my pain has gone why should I continue to come?” …I try to tackle and address that straight away” |
|  | Content  If you look at the text highlighted in yellow, it actually appears that there might be some confusion over the ’modalities’ used in MC. | “….it´s also to ask about their health and well-being in general….it has to be part of it….. you have to “educate” them a bit to make them tell you these things…..we actually do care if they have had a problem with their stomach”  “...it makes up a package. I don´t think it makes sense to only consider peoples back problems, you have to take into consideration how they live, right...”  “…to help them remember what they can do themselves…that´s a big part of the MC treatment”  “…those I consider to have a great risk of recurrence I would probably recommend to workout on a regular basis instead of suggesting MC” |
|  | Frequency of care | “…upon (re)solution of their initial/acute problem I have told quite a few patients that I find it beneficial for them to get into MC treatment, and they respond that they feel confident that they can “control it” themselves and wish to call the clinic when they get any of their well-known symptoms and that´s perfectly fine with me…”  “… if they are able to react on their symptoms before a regular relapse, well in my book that´s maintenance as well..”  These two quotes illustrate one of two strategies. Surely there must be strategies illustrating the regular scheduling as well. |
|  | Termination of care | “…even if they get their treatments for free it is my experience that they don´t want more treatment than they actually need”.  “…when I think of the patients who have stopped their MC treatment… it´s gone well for a while, but then they come back… sometimes they even say things like: “Why did you let me go without treatment for so long!!” and then we are back with the MC treatment”.  “…tired of those who think they are to come here once a month…they are not allowed to do that”  “…I also have patients who come more often than I think they should…you try to increase the interval between treatments all the time but…!” |
| Significant professional considerations | Ethics-related considerations | “…one should not pressure patients into MC treatment if they have no need and I think that some people don´t have that need…”  “…we can´t just tell people to come back once a month, I know a lot of chiropractors around the world, their maintenance programs are 3-4 weeks regardless…”.  “…it must not just be a convenient thing for me, that´s not right in an ethical sense, not even if people ask for it themselves, there has to be an actual and real need….”  “…coming in every three months on a regular basis without having symptoms… I can´t make myself practice that way….”  “…it´s “un-danish” this thing to plan long schematic treatment courses…”  “…I don´t want to be known as someone where it´s said that: “you have to come here for the rest of your life”…”  “… I have seen mr. X himself in England… completely unethical if you uncritically plan for every patient to come back for many many treatments…”  “…it has to be based on the given situation… otherwise we risk ending up as in USA where 60 treatments can easily be planned on your first visit…”  “… it may very well be that I “cheat” some of my patients…. I can´t deny that some of my patients might benefit from MC but don´t get it….”  “… some people practice a lot more MC than I do…. I don´t know if that is out of sheer habit but there is of course also an economical aspect of this…I think MC is being used quite a lot here and there….”  “… if I could sell this product –“it is absolutely necessary for your health that you come here once a month for the rest of your life”, I guess people would come. Many are very skilled in this, I´m just not…”  “… clearly some will do it out of economical reasons alone – others will find that this is not okay and then they forget that there might be other reasons than money to place patients on MC.”  “… even though I have patients who get 100% insurance paid treatment …I don´t think they would come here for no reason…” |
|  | Professional practice considerations | “..I had no experience with MC whatsoever when I started”  “…it was not at all on the schedule at school – we didn´t even talk about it!”  “….I just found out myself that it works…”  “… the time spent in the same clinic for my part has been of essence… to be able to follow the same persons for years”  “… I took over a clinic where a lot of MC treatment was given… too much!”  “… Maintenance is an individual solution… this is part of our unique product”.  “… some are kind of very much against the use of MC and some are very much for it…but on the bottom line there are patients who want one thing and patients who want another, so it seems reasonable enough to offer different things”  “…it´s very much those who don´t use MC… they are almost angry at those who do….but on the other hand some of the MC-chiropractors think that the others simply let down their patients for instance when they don´t bother to have training facilities, I for one find that absolutely wrong”  “… whether you think people should do as much as possible or as little as possible, you should just decide that with your patient right…?”  “… professionally some of the MC patients are actually quite boring to treat, it´s not a big challenge”. This indicates that MC in one-dimensional.  “… you have to be careful about the MC patients, some of them trust you to be able to treat every kind of problem…also always be aware that they might develop other problems or get diseases as for instance cancer”.  “…. It would be nice… learn more about it in the beginning or at least be more aware of what factors are of importance”  “… the problem is that I have not been able to identify which patient is to be put in which box!”  “… we would like some investigation of whether there is a…function-related outcome for the patient. Or …if the society, in an economically perspective, benefits from the fact that patients are being treated with MC”. |
